# Near-infrared light increases ATP, extends lifespan and improves mobility in aged *Drosophila melanogaster*

**DOI:** 10.1098/rsbl.2015.0073

**Published:** 2015-03

**Authors:** Rana Begum, Karin Calaza, Jaimie Hoh Kam, Thomas E. Salt, Chris Hogg, Glen Jeffery

**Affiliations:** 1 Institute of Ophthalmology, University College London, London EC1V 9EL, UK; 2 Program of Neuroscience, Institute de Biologia, Universidade Federal Fluminense, Rio de Janeiro 24210130, Brazil; 3 Moorfields Eye Hospital, London EC1V 2PD, UK

**Keywords:** lifespan, ATP, fly, inflammation

## Abstract

Ageing is an irreversible cellular decline partly driven by failing mitochondrial integrity. Mitochondria accumulate DNA mutations and reduce ATP production necessary for cellular metabolism. This is associated with inflammation. Near-infrared exposure increases retinal ATP in old mice via cytochrome *c* oxidase absorption and reduces inflammation. Here, we expose fruitflies daily to 670 nm radiation, revealing elevated ATP and reduced inflammation with age. Critically, there was a significant increase in average lifespan: 100–175% more flies survived into old age following 670 nm exposure and these had significantly improved mobility. This may be a simple route to extending lifespan and improving function in old age.

## 1. Introduction

Mitochondria provide cellular energy via adenosine triphosphate (ATP). But, their DNA (mtDNA) suffers from progressive mutations resulting in reduced ATP production, which is thought to run concomitantly with an increase in pro-inflammatory reactive oxygen species (ROS) [1,2]. Hence, hallmarks of ageing are reduced cellular energy and progressive systemic inflammation. Metabolic demand also plays a role as tissues and organisms with high metabolic rates generally suffer from rapid ageing [3,4]. The retina has the greatest metabolic demand in the body [5], but ATP decline in the central nervous system can be significantly improved by near-infrared/infrared light (NIR/IR, [6]). Specific wavelengths in this range are absorbed by cytochrome *c* oxidase in mitochondrial respiration, improving its efficiency [7–10]. These wavelengths improve mitochondrial membrane potentials, significantly reduce inflammation and reduce macrophage numbers with brief exposures of around 60–90 s repeated over approximately a week [11,12]. NIR/IR also reduces experimental pathology when insult impacts on mitochondrial function, as in experimental Parkinson's disease, where NIR significantly reduces cell death in the substantia nigra [13]. However, NIR/IR studies have largely used light for short periods and their impact on lifespan has not been assessed [7,11,12]. If NIR improves mitochondrial function we predict it may extend life. The fly has been used here because of its relatively short life [14]. Hence, we ask if long-term exposure to 670 nm in *Drosophila melanogaster* can increase lifespan and improve function in old age.

## 2. Material and methods

*Drosophila melanogaster* were used. Hatched male flies were housed on 12/12 light cycle at 25°C within a season. Half were exposed to 670 nm for 20 min per day at 40 mW cm^−2^ in clear plastic 50 cm^3^ (28 mm wide) containers, illuminating flies from either side, which were counted weekly. Room illumination was 2 mW cm^−2^. 670 nm energies were approximately 100 times lower than indirect sunlight, consistent with earlier studies [7]. Light devices were built by C. H. Electronics UK and contained 50 670 nm LEDS over 20 cm^2^. Six independent replicates were used in lifespan experiments (*n* = 620 flies). ATP, inflammation and mobility were assessed at seven weeks, when ATP and mobility are known to decline [15].

ATP was measured by luciferin–luciferase assay (Enliten^®^ ATP Assay System, Promega). Flies were killed with liquid nitrogen, transferred to 2.5% trichoroacetic acid (TCA), then homogenized at 4°C. Supernatant was collected and the TCA was neutralized with 1 M Tris–acetate buffer (pH 7.75, final TCA concentration 0.0625%); 10 µl of neutralized solution was added to 100 µl of luciferin–luciferase in fresh buffer. ATP was measured using an Orion microplate luminometer (Berthold Detection Systems GmbH) and data normalized to fly numbers.

Tissues were homogenized in 2% sodium dodecyl sulfate (SDS) with protease inhibitor cocktail for Western blot (Roche Diagnostics), and centrifuged; the supernatant was pipetted out, separated with 10% SDS–PAGE and electrophoretically transferred onto nylon membranes. Immunoblotting was undertaken for complement component C3 (Cappel, MP Biomedicals), which is highly conserved [16]. Protein was quantified by densitometric X-ray scanning and values were normalized to α-tubulin.

Fly mobility assessment was as Bjedov *et al.* [14]. Flies were placed in 100 ml clear cylinders (seven flies per trial), tapped to the bottom and then videoed, the last two steps repeated three times. Using the videos, the number of flies above the 50 ml mark (9 cm from the bottom) was counted after 1 min. Individual flies were traced, with absolute distance travelled measured.

Data were analysed with GraphPad Prism v. 5 and statistical analysis was undertaken using Mann–Whitney *U* non-parametric and log-rank tests.

## 3. Results

### (a) ATP levels are elevated and systemic inflammation reduced

**Figure 1.**
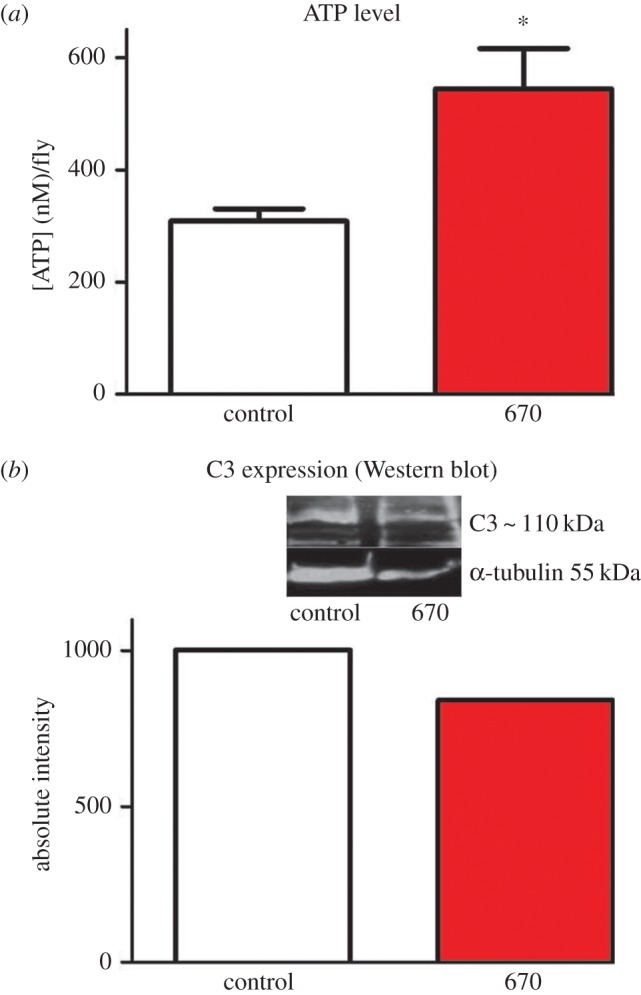
Exposure to 670 nm radiation increases ATP in aged flies and reduces inflammation. (*a*) Seven week old flies exposed to 670 nm had a significant increase in whole body ATP compared with controls, *p* = 0.028. *n* = 25 flies per group. (*b*) Whole body inflammation (C3) was measured in seven week flies using Western blot. This was reduced in 670 nm exposed flies by approximately 15%. Here, flies were pooled within groups as C3 protein levels were low in individuals. Hence there are no error bars. *n* = 15 flies per group. (Online version in colour.)

Whole body ATP declines with age only after approximately seven weeks [14], when ATP was measured here. ATP concentrations were significantly greater, by approximately 80%, in 670 nm exposed animals compared with unexposed (figure 1*a*, Mann–Whitney test *p* = 0.028). At seven weeks, Western blots were undertaken for inflammatory marker complement component C3. This was reduced in 670 nm exposed flies compared with controls (figure 1*b*). Hence, 670 nm radiation elevates ATP and reduces inflammation.

### (b) Lifespan increases

**Figure 2.**
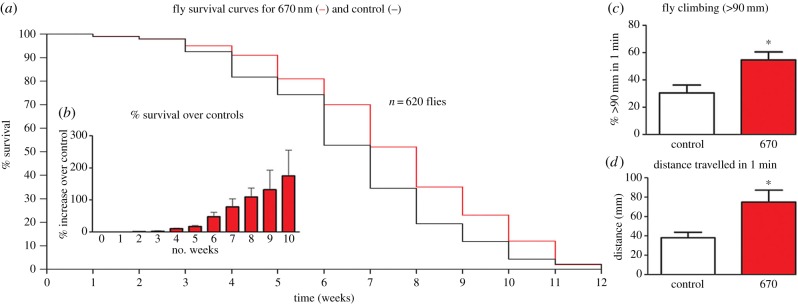
Lifespan and mobility. (*a*) Fly numbers at progressive weeks in groups exposed to 670 nm supplemented light each day (red line) and controls (black line). Curves are averages for six independent experiments with a minimum of 40 flies per group in each experiment. Fly death rates separated between three and six weeks with fewer flies dying in 670 nm exposed animals. Reduction in the two population followed similar patterns from six weeks but with the 670 nm exposed group having greater numbers at any point until week 12. In all replicates, there was no indication that 670 nm increased absolute lifespan beyond weeks 11–12. Differences between the two groups were statistically significant (*p* = 0.008). (*b*) Inset: percentage increase of 670 nm exposed flies alive at progressive weeks. (*c*) Seven week old 670 nm exposed flies were more active than controls. (*d*) Mobility measures the percentage of flies that climbed above 90 mm in a clear 100 ml cylinder. (*d*) This was filmed and then the distance travelled by each fly was measured in each group. In both cases, the 670 nm exposed flies where significantly more mobile. There were 21 flies in each group in each condition. (Online verion in colour.)

Fly numbers in experimental and control groups were similar in the two weeks post-hatching. From week 3, fly deaths were greater in controls than 670 nm exposed flies and they remained so at each time point until week 11–12, when all flies were dead in both groups. This difference was significant (figure 2, log-rank test *p* = 0.008).

The progressive mean percentage increase in 670 nm flies alive over controls is given in figure 2*b*. Group differences accelerated from week 4, when 10% extra 670 nm treated flies were alive compared with controls, to approximately 50% extra when the control population had halved. By the time the control population was reduced by 80%, at week 8, more than 100% extra 670 nm treated flies remained alive. Subsequently, group differences reached almost 180% before declining to zero in both groups at week 11–12. Hence, 670 nm did not extend absolute lifespan.

### (c) Aged mobility increases

Mobility of 670 nm treated and control flies was measured at seven weeks. Significantly more 670 nm treated flies climbed above the 50 ml level (9 cm) and significantly more travelled a greater distance than controls (Mann–Whitney test *p* = 0.028, *p* = 0.014, respectively). Twice as many 670 nm flies climbed above 50 ml (9 cm) compared with controls and these travelled twice the distance in 1 min compared with controls (figure 2*c,d*). Hence, 670 nm exposure significantly improves both lifespan and mobility.

## 4. Discussion

*Drosophila melanogaster* has been widely used in lifespan studies as they are short lived and their genomic sequence is relatively well understood [14,17], hence their adoption experimentally here to extend lifespan. Our results reveal that when flies are exposed to 670 nm radiation they have reduced inflammation, improved ATP, improved mobility and extended average lifespans. These data are consistent with the majority of studies undertaken using 670 nm on mammals, showing reduced inflammation in experimental models and in ageing, and improved ATP levels [6,7]. However, it would be difficult to undertake lifespan experiments in mice as the light would not penetrate the entire body as it does in flies and hence its influence would not be systemic.

There are many factors and pathways in ageing, and nine candidate hallmarks have been suggested, which may be separate, but also are likely to have interactions [1]. Mitochondrial function is one. Previously, mitochondrial function and ageing were viewed within a framework of progressive mtDNA mutations/deletions resulting in reduced ATP and increased ROS. The balance of these factors was seen as a driver in the mitochondrial theory of ageing [18]. However, evidence has undermined the role of ROS in ageing [19,20]. Hence, some mutant mice have reduced lifespan as a result of mtDNA mutations/deletions not associated with increased ROS [21,22]. Further, increased ROS can prolong lifespan in yeast and *Caenorhabditis elegans* [22,23], and in mammals it does not accelerate ageing [20]. These data are reviewed by Lopez-Otin *et al*. [1], who argue that low ROS may activate compensatory mechanisms and not directly contribute to ageing. Such data may undermine the ROS element in Harman's mitochondrial theory [18]. If correct, it places greater potential emphasis on ATP in ageing.

NIR has been successful in treating induced pathology [7] and ageing, particularly in the retina, where progressive age-related inflammation is marked owing to high metabolic rate [11,12]. These wavelengths penetrate deeply and 670 nm trans-illuminated our flies at 40 mW cm^−2^. In relation to this, it may be significant that, while old domestic incandescent lighting contained significant NIR elements, none is present in modern strip lighting or energy-saving domestic lighting [12]. The absence of these wavelengths from artificial lighting may have long-term consequences. As longer wavelengths penetrate deeply, this may be of significance not only for the ageing eye, but also potentially for other tissues.

## Data accessibility

All data are presented in the manuscript.
